# LB7. Ad26.COV2.S-Elicted Neutralizing Activities Against SARS-CoV-2 Variants of Concern in Phase 1/2a and Phase 3 Clinical Trials

**DOI:** 10.1093/ofid/ofab466.1638

**Published:** 2021-12-04

**Authors:** Mathieu Le Gars, Jerald Sadoff, Mandy Jongeneelen, Dirk Heerwegh, Georgi Shukarev, Carla Truyers, Anne Marit de Groot, Gert Scheper, Jenny Hendriks, Boerries Brandenburg, Frank Struyf, Johan Van Hoof, Macaya Douoguih, Hanneke Schuitemaker

**Affiliations:** 1 Janssen Vaccines and Prevention, Leiden, The Netherlands, Leiden, Zuid-Holland, Netherlands; 2 Janssen Infectious Diseases and Vaccines, Leiden, Zuid-Holland, The Netherlands, 2333, Leiden, Zuid-Holland, Netherlands; 3 Janssen Research and Development, Beerse, Belgium, Beerse, Antwerpen, Belgium; 4 Janssen Vaccines and Prevention, Leiden, Netherlands, Leiden, Zuid-Holland, Netherlands

## Abstract

**Background:**

In a Phase 3 trial, the Janssen COVID-19 vaccine, Ad26.COV2.S, showed robust efficacy against severe–critical COVID-19 in countries where different SARS-CoV-2 variants were circulating. We evaluated Ad26.COV2.S-elicited antibody neutralizing activity against variants of concern (VOC) B.1.1.7 (Alpha), B.1.351 (Beta), and B.1.617.2 (Delta) in sera from participants in clinical trials following a single dose of Ad26.COV2.S.

**Methods:**

Neutralizing activities of Ad26.COV2.S (given at a dose level of 5 x 10^10^ viral particles [vp]) against VOC were assessed by wild-type virus neutralizing (wtVNA) and pseudovirion neutralization (psVNA) assays in sera from participants in Phase 1/2a and Phase 3 clinical trials, respectively. Geometric mean titers (GMTs) were determined at Days 29 and 71 after vaccination.

**Results:**

In serum samples from Phase 1/2a participants (n = 6), at Day 29 after 1 dose of Ad26.COV2.S, wtVNA titers against VOC were lower than for the original strain (GMT = 573), with GMT = 65, 14, and 15 for Alpha, Beta, and Delta, respectively, representing 8.8-, 40.9-, and 37.7-fold decreases. By Day 71 after vaccination (n = 14), fold differences between the original strain (GMT = 375) and VOC (GMT = 113, 27, and 28) were smaller (3.3-, 13.9-, and 13.4-fold) than at Day 29, suggestive of B-cell maturation (**Figure 1**). Day 71 titers against the Delta variant were maintained for at least 8 months following a single dose of Ad26.COV2.S (5 x 10^10^ vp). In serum samples from Phase 3 participants (n = 8), psVNA titers against VOC were lower than the original strain at Day 71 after vaccination, with the lowest titers observed for the Beta variant (3.6-fold decrease vs original strain). Smaller reductions in Nab titers for VOC were observed in the psVNA assay compared to wtVNA.

Figure 1. Neutralization of B.1.1.7 (Alpha), B.1.351 (Beta), and B.1.617.2 (Delta) lineages in serum samples from participants who received Ad26.COV2.S. n = 6 samples at Day 29 and n = 14 (n = 14 for Alpha and Beta; n = 6 for Delta, comprising the same 6 participants at Day 29) samples at Day 71 after vaccination with a single dose of Ad26.COV2.S (5 x 10^10 vp dose level) were analyzed in wild-type virus neutralization assays against the SARS-CoV-2 Victoria strain (D614, black dots), the B.1.1.7 (Alpha; green dots) the B.1.351 (Beta; blue dots), and the B.1.617.2 (Delta; purple dots) lineages. Dots represent the IC50 (inhibitory concentration) titers per participant. Geometric mean titers (GMTs) and fold decrease in neutralizing activity between the original Victoria strain and each lineage are shown.

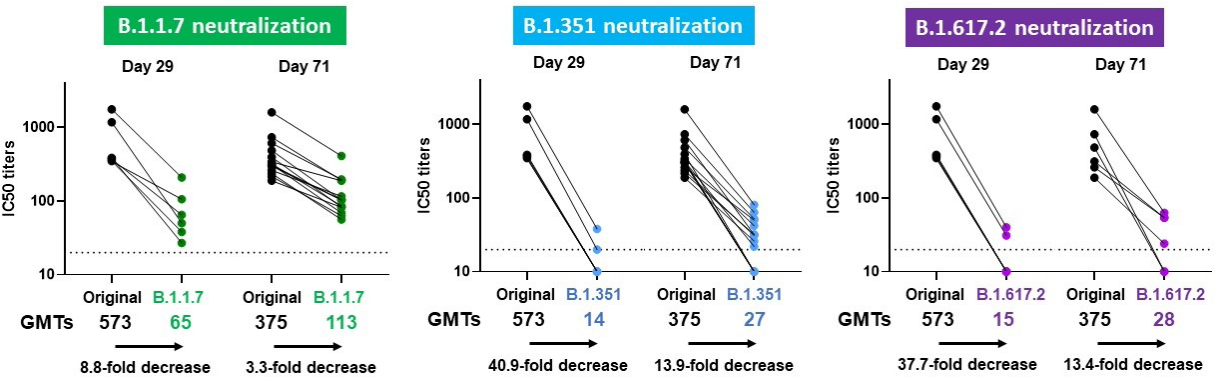

**Conclusion:**

Ad26.COV2.S-elicited serum neutralizing activity against VOC showed an overall decrease in titers relative to the original strain that was largest for the Beta variant, even though vaccine efficacy against severe–critical COVID-19 was maintained in countries where these variants were circulating versus in countries where they were not circulating. Over time, titers against variants increased, suggesting B-cell affinity maturation leading to increasing coverage of VOC.

**Disclosures:**

**Mathieu Le Gars, n/a**, **Johnson & Johnson** (Employee, Shareholder) **Jerald Sadoff, MD**, **Johnson & Johnson** (Employee, Shareholder) **Mandy Jongeneelen, n/a**, **Johnson & Johnson** (Employee, Shareholder) **Dirk Heerwegh, n/a**, **Janssen Research and Development** (Employee) **Georgi Shukarev, MD**, **Janssen** (Employee) **Carla Truyers, n/a**, **Janssen Research and Development** (Employee) **Anne Marit de Groot, n/a**, **Johnson & Johnson** (Employee) **Gert Scheper, n/a**, **Johnson & Johnson** (Employee, Shareholder) **Jenny Hendriks, n/a**, **Johnson & Johnson** (Employee, Shareholder) **Boerries Brandenburg, n/a**, **Johnson & Johnson** (Employee, Shareholder) **Frank Struyf, n/a**, **Johnson & Johnson** (Employee, Shareholder) **Johan Van Hoof, n/a**, **Johnson & Johnson** (Employee, Shareholder) **Macaya Douoguih, MD, MPH**, **Janssen** (Employee) **Hanneke Schuitemaker, PhD**, **Johnson & Johnson** (Employee, Shareholder)

